# Dr. Thomas Kieth

**Published:** 1895-12

**Authors:** 


Dr. Thomas Kieth, of London, died at his home, October 9, 1895, aged 68 years. He received his early education at Aberdeen, but his medical education was entirely conducted at Edinburgh, where he lived during the greater part of his professional life. He served as an apprentice to Sir James Y. Simpson and married a first cousin of Simpson's wife. In his first professional years he practised obstetrics with conspicuous success, yet at an early period he devoted himself to general surgery, but later limited his practice entirely to gynecological surgery. In 1862, Kieth performed his first ovariotomy, at which time Sir Spencer Wells had done but eight such operations.

Believing the general hospitals were not sufficiently aseptic he reconstructed a house in Edinburgh at a cost of $15,000, that served as a private hospital where many of his early operations were undertaken. In 1878, he published statistics that astonished the world on account of the low rate of mortality that they exhibited. In 1888, he moved to London, where he has since resided, and in association with his son, Mr. Skene Kieth, continued the practice of abdominal surgery. Of late years he turned his attention to the electrical treatment of fibroids, in which he was somewhat enthusiastic, but it is doubtful whether his hopes and expectations have been fully realised. For the last three years Dr. Kieth's infirmities prevented him from doing any active work. During the past summer he visited his home near Montrose, in Scotland, where he somewhat improved, but on returning to London the heat proved debilitating and his disease terminated fatally Wednesday morning, October 9th. He was buried on Friday in Kensal Green Cemetery. Dr. Kieth was an honorary fellow of a large number of British and foreign societies and, in the United States, where he was deeply respected, his memory will long remain green.

